# The New Year

**Published:** 1916-01

**Authors:** 


### EDITORIAL DEPARTMENT

MRS. F. E. DANIEL, Publisher and Managing Editor.
DR. R. H. L. BIBB, Associate Editor.

CONTRIBUTING EDITORS:

Dr. H. Sheridan Baketel, New York, editor *Medical Times:* Dr. M. H. Boerner, Austin, Texas; Dr. G. Henri Bogart, Paris, Ill.; Dr. M. M. Carrick, Dallas, Texas; Dr. Edward H. Carey, Dean Medical Department, Baylor University, Dallas, Texas; Dr. W. L. Crosthwaite, Waco, Texas; Dr. T. D. Crothers, Hartford, Conn.; Dr. H. W. Cummings, Hearne, Texas; Dr. Oscar Dowling, New Orleans, President Louisiana State Board of Health; Havelock Ellis, M. D., London, Eng.; Dr. May Agnes Hopkins, Dallas, Texas; Dr. A. W. Fly, Galveston, Texas; Dr. G. B. Foscue, Waco, Texas; Dr. E. S. Goodhue, Holualoa, Kona, Hawaii; Dr. M. B. Grace, Seguin, Texas; Dr. Joe Gilbert. Austin, Texas; Dr. Charles H. Hughes, St. Louis, editor *Alienist and Neurologist*; Dr. James G. Kiernan, Chicago; Dr. George H. Lee, Galveston, Texas; Dr. John T. Moore, Houston, Texas; Dr. Frank Paschal, San Antonio, Texas; Dr. John Preston, Austin, Texas, Superintendent State Lunatic Asylum; Dr. G. Wilse Robinson, Kansas City, Mo.; Dr. Bacon Saunders, Fort Worth, Texas; Dr. Allen J. Smith, Philadelphia, Medical Department, University of Pennsylvania; Dr. Z. T. Scott, Austin, Texas; Dr. Ralph Steiner, Austin, Texas, President Texas State Board of Health; Dr. Walter S. Sutton, Kansas City, Mo.; Dr. A. E. Sweatland, Nacogdoches, Texas; Dr. John S. Turner, Dallas, Texas; Dr. Charles Venable, San Antonio, Texas.

---

### The New Year.

It seems but a few months since the TEXAS MEDICAL JOURNAL was giving a New Year's greeting to its patrons; twelve months roll around quickly when one is busy, or when one is getting old. The editor of the JOURNAL has been busy, and the JOURNAL itself is old for a publication of its kind, and this combination of employment and age may account for the unusual rapidity with which the year has passed.

A year ago, with but a few months of experience as the responsible manager of a publication for the medical profession, the editor of the TEXAS MEDICAL JOURNAL spoke with hesitation of the efforts she proposed to make for its improvement. There was no doubt on her part as to her determination to make the JOURNAL better, but the doubt and hesitancy was due to a lack of confidence in herself,—in her ability to make good. She has worked earnestly and faithfully to redeem the pledge made then, and at times the effort has seemed more than her physical and mental endurance could stand, but she has at no time faltered. After all, it is not so much what one accomplishes that counts; it is what one tries to do that measures success. This thought has consoled when mistakes have been made and failures have been encountered.

That the promise of a year ago has been at least fulfilled in part has been shown by the words of cheer and commendation that have been received from all sources. Physicians by the score have said or have written that they enjoy the TEXAS MEDICAL JOURNAL, and get much good from it; advertisers have given assurance that they have found their investments in it profitable to them. The business has shown a steady increase every month of the year, and the number of subscribers has steadily grown. This has not come without hard effort, however, for, in this day of strong competition, merit must exist for a publication to succeed. The TEXAS MEDICAL JOURNAL has never appealed for sympathetic support from either subscribers or advertisers; it would not knowingly accept business for sympathetic reasons; the publication that does not deserve to live deserves death.

The editor is not vain enough to think that the almost phenomenal success of the past year, under great disadvantages, has been due solely, or even largely, to her efforts, except insofar as she has endeavored to make and to merit the friendship of the members of the profession, who have rendered such valuable assistance. The very best men in the profession in Texas and in other States have contributed editorials and papers that have attracted the attention of eminent medical men and surgeons all over the country, and have directed the leading professional minds to the high standing of the TEXAS MEDICAL JOURNAL. Without these contributions the JOURNAL could not have succeeded, and the editor wishes to use this opportunity to render her grateful thanks to every friend who has thus given her necessary aid, and to solicit their further favors in this way. Those who have been regular readers of the JOURNAL have told other physicians of their regard for and interest in it, and have thus aided in increasing the circulation. Thanks are returned for every kind word spoken in its behalf, and to every subscriber who has taken and read the publication. The subscribers to the MEDICAL JOURNAL are "the cream" of the profession in Texas and other States, the men who study and bring about the scientific advances in medicine and surgery; they are the picked men who are doing the constructive work—the leaders in the profession.

The advertising has increased despite the plaint of "hard times." Advertisers have been courteous and business-like in their dealings, and have shown in many ways their good-will. They have profited by using the pages of the TEXAS MEDICAL JOURNAL, and in a number of cases have increased their advertising space—because they have found it paid them to do so. Thanks are rendered them, and they are assured that every effort is made to give big value for every dollar spent with the publication.

Now, as to the coming year; we can only say that, encouraged by the results of the past year, still stronger efforts will be made to improve the TEXAS MEDICAL JOURNAL in 1916. Experience is worth much, and this experience will be used for the benefit of every reader and every advertising patron. This is an age of advancement, and especially is advancement seen in medicine and surgery. The JOURNAL expects to keep step, and to enable its readers to keep step. It can't afford to rest on the accomplishments of the past any more than you can afford to do so. Let's co-operate for our mutual advantage. It is the earnest effort of this editor—the everyday effort—to be of some service, and especially to serve those who by their assistance make it possible for her to serve. May 1916 be the best, the most useful year for each of us, and may the wish inspire the effort to make it so.

---

There are too many physicians who depend upon the letters following their names more than upon their study and understanding of medical science. Degrees and titles have never yet assured success. They only indicate that a professional man has reached the point where he ought to be able to advance, provided he applies himself diligently enough.

---

Whatever you may wish about it, the physicians in a generation or two are going to be devoting their time more to preventive measures than to diagnosing and treating diseases. The people are learning that it is better to stay well than to get sick and employ physicians to cure them. Physicians will be in demand just as much as now, but they will in most cases begin their diagnosis and treatment before their patients get down with disease.

---

The city that neglects to take the necessary sanitary measures for safeguarding the public health does not deserve to grow. We are horrified beyond measure at the ravages of war but take little note of the diseases and pestilences that are due to official neglect. Of course, when an epidemic prevails, cities and States become immensely alarmed, but that is too late to obtain the best results. The people are under obligations to themselves to have the best health regulations and sanitary measures, and this obligation extends to rich and poor alike.

---

The physician of this generation has almost forsaken the practice of a decade ago of spending a time at the bedside of the patient, talking with the family cheerfully and encouraging patient and friends to hope for an early recovery. The old family doctor, after diagnosing a case and prescribing and perhaps giving the medicine, made somewhat a social event of his call. Almost invariably his visit relieved the tense fears of the family as to the condition of the patient, and this aided the patient. Now physicians have caught the hurry of the age and, whether they are busy or not, hasten away from the home of sickness as though they were leaving some plague spot. It is true that many of them are busier than the old family doctor with his pillbags used to be, but even when they have the time they seldom tarry a minute longer than necessary. At the risk of being classed as a stickler for obsolete practices, the suggestion is made that the old custom of remaining awhile for a visit at the bedside is still appreciated by most people.

---

The Medical Package Library inaugurated a few months ago by the TEXAS MEDICAL JOURNAL is steadily growing in favor with the progressive physicians and surgeons of the Southwest. It is because it meets a demand for quick information on subjects that are constantly arising in the profession, and saves its patrons many hours of tiresome and often fruitless research. If you want to keep right up with the medical literature that will be helpful to you, write for information about the Medical Package Library.

---

It is hoped that the newspapers, magazines and medical journals of the country have said all they care to say about the Bollinger baby case. It should never have been given any publicity unless it had been in the medical journals for the purpose of directing the thought of other physicians to their obligations under like conditions. To use it for purposes of personal exploitation, as has been charged, is the basest kind of commercialism.

---

The physician has an unusual influence in his community if he will. In the first place, his education is way above the average; he is called into friendly and confidential counsel by many if he deserves their confidence; he should be a safe adviser because of his intellectual equipment. He should be a leader among men, and not solely a physician and healer to the sick.

---

El Paso is to have a new modern hospital, which will be equipped with all modern conveniences. Mrs. W. M. McDonald has awarded the contract, and work will begin as soon as the material can be assembled.

